# Microstructure and phases of deposited metal of SUPER304H steel under high temperature Persistent stress

**DOI:** 10.1038/s41598-018-20594-9

**Published:** 2018-02-08

**Authors:** Yanchang Qi, Zhiquan Wu, Xin Zhang, Chengyong Ma

**Affiliations:** 1 Welding Research Institute, Central Iron and Steel Research Institute, Beijing, 100081 China; 2 Institute of New Energy Research, China Datang Coporation, Beijing, 100040 China; 3 Institute of Thermal Power Generation Technology, China Datang Coporation Science and Technology Research Institute, Beijing, 100040 China

## Abstract

In order to investigate the high temperature rupture property of deposited metal of SUPER304H steel, the high temperature tensile test was carried out, and the microstructure transformation of deposited metal of SUPER304H steel under high temperature persistent stress were studied. Most of the solidification subgrain boundaries dissolve. The effect of the high temperature enduring on the microstructure is not obvious. Temperature and time are the main factors that influence the change of microstructure. Under the action of high temperature stress, the corrosion resistance of austenite decreases significantly due to the occurrence of chromium deficiency. With the persistent stress of 200 MPa, the precipitated phase of deposited metal is Nb (C, N), M_23_C_6_, NbCrN phase, and a certain amount of alpha phase is precipitated in the deposited metal with a persistent stress of 78 MPa. The precipitation of M_23_C_6_ phase is the main reason for the decrease of the corrosion resistance, especially the decrease of the corrosion resistance.

## Introduction

In order to meet the requirements of energy saving and environmental protection, and realize the high efficiency of thermal power generation, the steam temperature and pressure raise higher requirements for boiler steel. For this, many scholars are constantly exploring ways to improve the performances of heat-resistant steel, and a series of heat resistant steels with good performance and high durability has been developed^1,2^, such as Super304H, T92, TP347HFG, and so on.

Super304H steel is widely used in the boiler superheater and reheater^3^. The main problems in Super304H steel welding are the hot cracking, corrosion and aging embrittlement^4,5^. The welding joints of Super304H steel have some differences between the weld and heat affected zone compared with the base metal, and these differences make the performance of welded joints different from that of base metal. In terms of precipitation strengthening, the change of size, quantity, shape and distribution of different parts of the welded joints is different, but the mechanism of the effect of these changes on the joint performance is rarely reported^6,7^. The precipitates in the heat affected zone of Super304H steel can precipitate, aggregate and grow, which will lead to the change of material properties^8^. In addition, there will be a softening zone and coarse grain zone in the weld heat affected zone. The mechanism of these changes is not very clear^9^. The welding method of Super304H steel is the gas tungsten arc welding. There is a big difference between the welding material and the base material, and the welding seam can not be treated as the parent material in the welding process^10^. Therefore, there are great differences between the microstructure of the weld and the base metal, such as the type of precipitates, the grain size and the crystal structure. Their influence on the performance is sometimes greater than that of the heat affected zone, which leads to the changes of the weld performance and corrosion. Due to the high degree of supersaturation after welding of Super304H^11^, there was more obvious reduction in the aging process, and there are some differences between the factors of aging embrittlement and the base metal. Therefore, it is necessary to study the cause of aging embrittlement. According to the published literature, the research mainly focused on the properties and strengthening mechanism of Super304H steel base material and the selection of the welding material^12^.

On the basis of the study of SUPER304H welding wire in the earlier period^13^, this paper has studied the microstructure and phases of deposited metal of SUPER304H steel under high temperature persistent stress in order to master the related properties of welding wire.

## Experimental Materials and Methods

### Experimental materials

The diameter of the welding wire was 1.6 mm. The actual compositions of the test welding wires were determined by ICP-AES analysis and the results were presented in Table 1. The test wire was used to deposite the cladding isolation layer with thickness of 8 mm on the groove surface and the pad surface, and the root clearance was 16 mm, which ensured that the deposited metal component was not affected by the dilution of the substrate. The SUPER304H austenitic heat-resistance steel is used for the test, and the composition of SUPER304H steel tube was shown in Table 2.

**Table 1:** Chemical composition of deposited metal (mass fraction, %).

Element	C	Si + Mn	Cr	Ni	Mo	N	Nb	Cu
content	0.080	3.39	16.97	15.24	1.09	0.13	0.28	2.91

**Table 2:** Chemical composition of Super304H (mass fraction, %).

C	Si	Mn	S	P	Cr	Ni	Mo	N	Nb	Cu	Co	Al
0.074	0.28	0.81	0.003	0.016	18.26	9.01	0.32	0.11	0.53	2.95	0.031	0.016

### Experimental Methods

Welding equipment adopted Manipulator AMET welding machine automatic TIG system. Welding heat input is 14 KJ/cm, and the specific welding process parameters are shown in Table 3.

**Table 3:** Welding process parameters of welding wire deposited metal.

Electric current/A	Voltage/V	Welding speed cm/min	Wire feeding speed mm/min	shielding gas	Gas flow L/min	Inter channel temperature □
260	12.7	14	800	Pure Ar	15	≤100

The test pressures were 200 and 78 MPa at the temperature of 650 °C. The sample spans SUPER304H weld and the center of the sample is the weld center. After grinding and polishing, the specimen was etched by the mixed solution consists of 5 g CuCl_2_, 30 ml HCl, 25 ml alcohol and 30 ml H_2_O. Then, the microstructure of deposited metal was investigated by using the MEF-4M metallographic microscope and SCIAS 6.0 image analysis system. The second phase of deposited metal and impact fracture surface were analyzed using Scanning Electron Microscope of HITACHI S-4300 with matching Energy Dispersive Spectrometer.

In order to confirm the possible precipitated phase, the method of electrochemical extraction was used to study the second phase of deposited metal. In addition, X Pert Pro X-ray diffractometer was also adopted for the analysis of phase composition of deposited metal.

### Determination of high temperature endurance parameters

The working temperature of Super304H steel pipe is 650 °C. According to the creep rupture data of Super304H steel pipe provided by A.Iseda *et al*.^14^, it is known that the fracture time of Super304H base metal at 200 MPa is about thousands of hours. Comprehensive consideration of the test results and the test cost, the high temperature endurance test of the deposited metal was carried out with 200 MPa as the persistent stress, and the durability of the deposited metal at the temperature of 650 °C was investigated.

## Results and Discussion

### Solidification subgrain boundary and Nb phase

**Figure 1:**
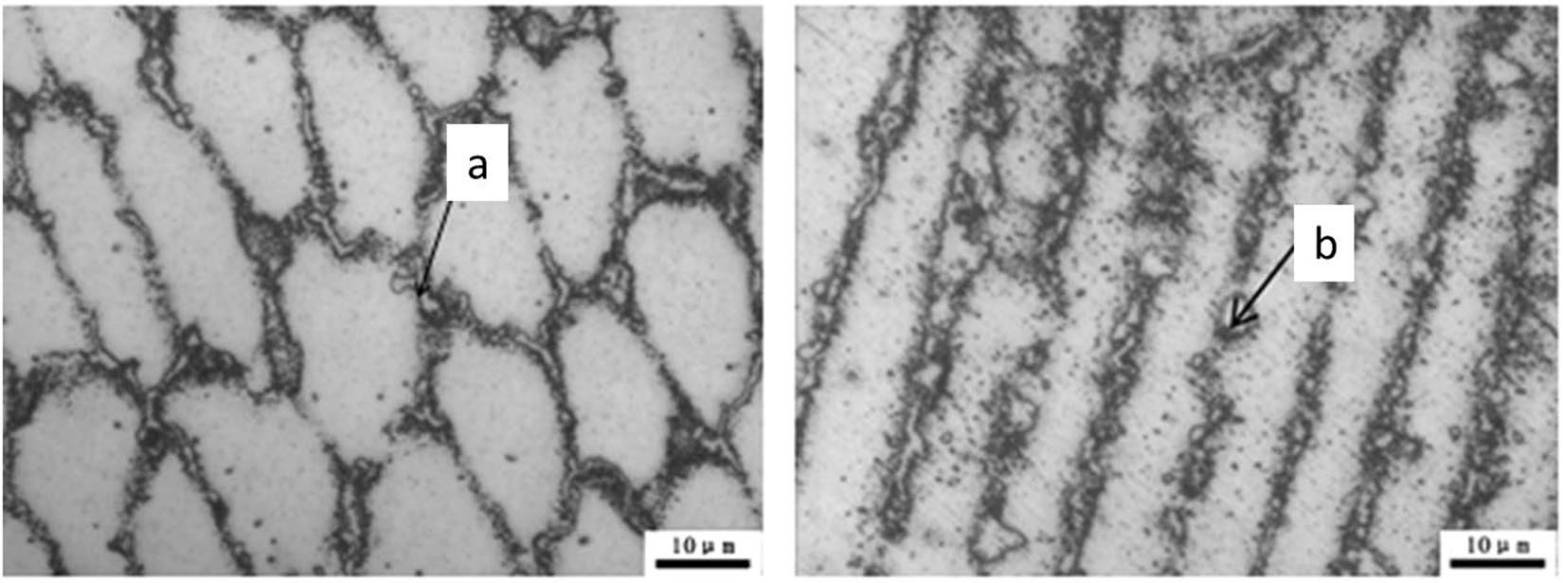
Different cross sections of the solidification subgrain boundary and Nb phase.

After subjecting to a persistent stress of 200 MPa and a lasting time of about 8960 h, the microstructure of the deposited metal is shown in Fig. 1. It can be seen that the niobium phases in the solidification subgrain boundaries of different sections have different degrees of dissolution. The partial positions of the niobium phases in the weld metal are dissolved, especially the edges and corners of the precipitates. Under the double action of high temperature and stress, Nb phase shape became round. The distribution of Nb phases presented the chain, bar and block shape, but the size change of Nb phases is not obvious and there is no obvious coarsening.

**Figure 2:**
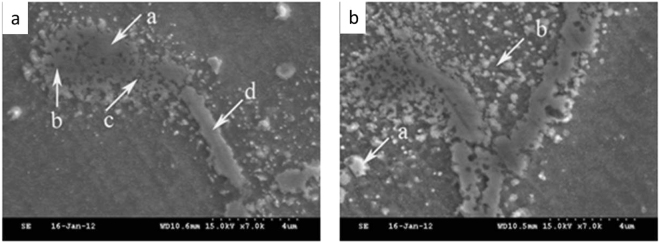
Typical SEM morphology of solidification subgrain boundary and Nb phase: a-location 1; b-location 2.

The two typical morphologies of Nb phase can be seen under SEM, as shown in Fig. 2. Niobium phases directly considered the austenitic matrix as the background, and the Niobium phase distributed with in the matrix with the chain or strip shape, as shown in Fig. 2(1)d. The Nb phase is distributed on the loose solidification subgrain boundary with the bulk shape, as shown in Fig. 2(1)a. After high temperature enduring-tensile tests, the solidification subgrain boundary changed obviously. After metallographic etching, the solidification subgrain boundaries in partial areas begin to dissolve, and the precipitates are directly embedded in the austenite matrix. However, the solidification subgrain boundaries become loose in other regions, and there is a certain degree of dissolution in the internal existed Nb phase. The micro morphologies of the solidification subgrain boundaries and Nb phase in location 1 of Fig. 2(1) was analyzed by energy spectrum. It is found that the lacation a in the solidification subgrain boundary is the dissolved Nb phases, and the interface between the Nb phase and the solidification subgrain boundary is not obvious, but the niobium content is still close to 19%, as shown in Table 4. The Nb content difference in the locations b and c which are close to location a is very small. From the point of view of composition, it is close to the composition of the weld solidification subgrain boundary, and it can be determined that the locations b and c are the solidification subgrain boundary, and the diffusion of niobium in niobium phase is very limited. The niobium phase in location d presents the strip shape, and the niobium content is up to 30%. The Nb content of the location d close to the niobium phase is 0.96%, which is very close to that of the solidification subgrain boundary. However, the Nb content of the location d is higher than that in the austenite matrix. It can be seen that the solidification of the sub grain boundaries in the location d have dissolved.

**Table 4:** Results of the analysis of the spectral components of the solidification subgrain boundary location 1 (mass fraction, %).

location	C	Si	Mn	Cr	Ni	Mo	N	Nb	Cu
a	0.06	0.62	4.32	20.39	12.94	1.75	0.14	18.91	4.51
b	0.04	0.69	4.75	22.62	16.71	2.05	0.10	0.92	5.23
c	0.05	0.44	4.29	20.96	15.17	1.71	0.16	1.20	5.59
d	0.06	0.13	4.03	19.74	9.38	1.58	1.21	28.02	4.76
matrix	0.04	0.38	4.30	18.23	14.31	1.06	0.07	0.96	4.25

The energy spectrum analysis is carried out in the region around the Nb phases of location 2 in Fig. 2(2). According to the analysis results of Table 5, the content of Cr, Ni, Mo and Nb in two loations is very close, which is slightly higher than that of the surrounding austenite matrix. Although the locations (a and b) showed different morphologies, both of them are solidification subgrain boundaries. It can be inferred from the different morphologies of the above solidification subgrain boundaries that the diffusion of elements in the fast diffusion channel will cause the decrease of the corrosion resistance during the high temperature lasting process.

**Table 5:** Results of the analysis of the spectral components of the solidification subgrain boundary location 2 (mass fraction, %).

location	C	Si	Mn	Cr	Ni	Mo	N	Nb	Cu
a	0.10	0.53	4.60	19.38	15.94	1.32	0.16	0.87	4.57
b	0.03	0.30	4.28	19.85	16.90	1.02	0.12	0.96	5.09

**Figure 3:**
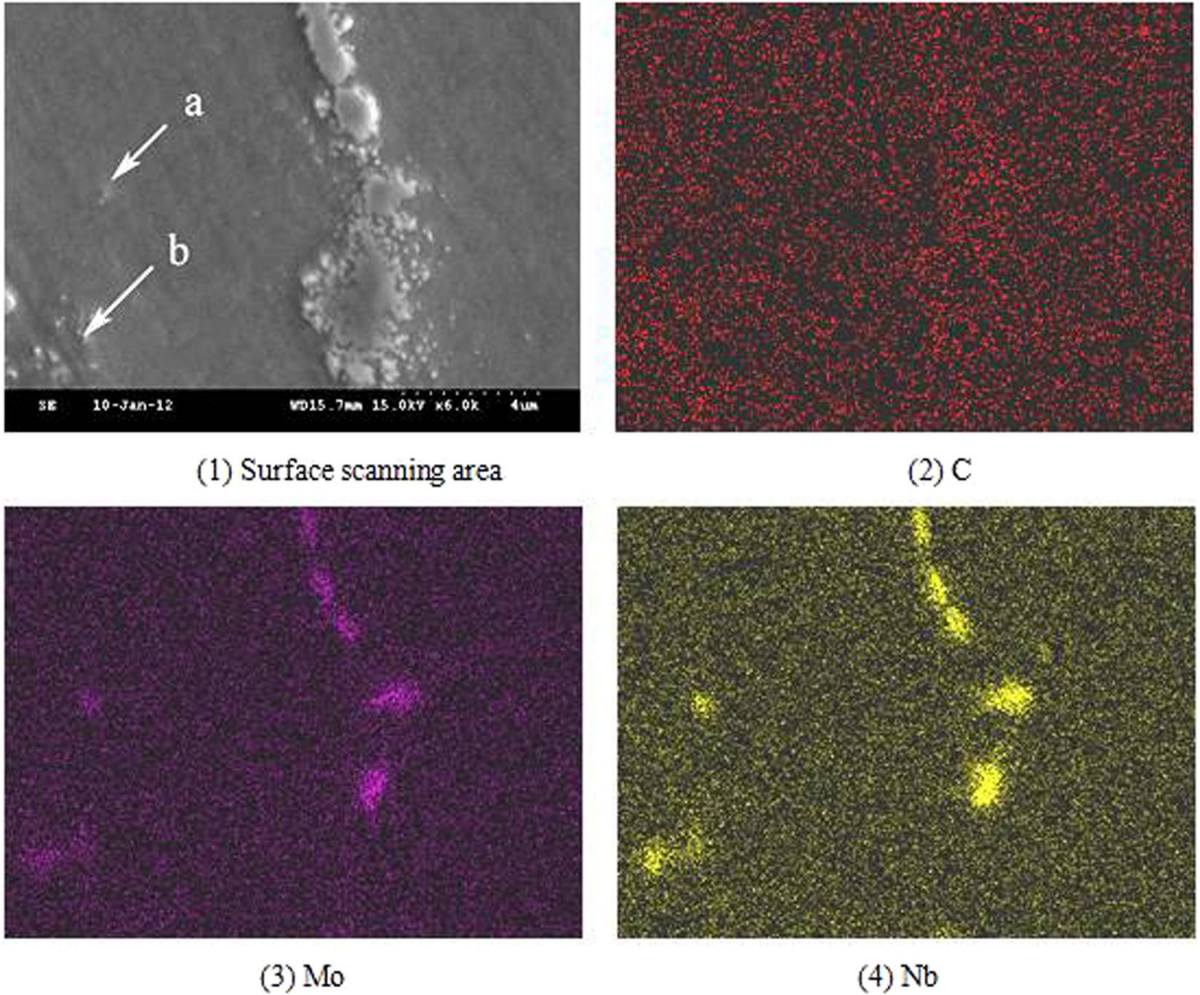
Scanning distribution of the Elemental Plane in the fully dissolved niobium phase.

Scanning analysis of the elements is carried out in the local region which was not influenced by the two welding thermal cycle in the deposited metal. Comparing with the scanning area and the element distribution, it can be found that the locations (a and b) are completely dissolved and almost completely dissolved in the Nb phase from the segregation of Nb and Mo elements in Fig. 3. Although the Nb phases in the two locations have lost the morphology of the phase, the Nb and Mo elements are still in the state of segregation, and no obvious diffusion has occurred.

The statistical analysis of the micro area compositions in the solidification subgrain boundary and the austenite crystal is shown in Table 6. Compared with the austenitic intergranular of the welded deposited metal, the content difference of alloying elements in austenite matrix before and after high temperature tensile elongation is small, and the content of niobium is only slightly higher, which may be related to the diffusion of the niobium element. Compared with the components of the solidification subgrain boundary in the weld metal, the contents of carbon, chromium and molybdenum increased obviously. The dissolution of niobium phase and the diffusion of molybdenum in the solidification subgrain boundary may lead to the increase of molybdenum content in grain boundary. The high temperature tensile process at the temperature of 650 °C is located in the sensitization zone, and M_23_C_6_ will precipitate in a wide temperature range with the effect of persistent stress, which is consistent with the conclusion of Beckitt and Clark^15^. Compared with the content of chromium in solidification subgrain boundary and austenite grain after high temperature enduring tensile, the content of chromium in the grain boundaries is about 4.2% higher than that in the matrix, but the content of chromium of the grain boundaries in the welded deposited metal is only about 1.2% higher than that in the matrix. Therefore, it can be inferred that the short distance diffusion of chromium from the matrix to the grain boundaries occurs. In addition, the partial dissolution of niobium on the solidification subgrain boundary can provide a certain amount of carbon element for the precipitation of M_23_C_6_. M_23_C_6_ has a complex cubic structure, which is easier to dissolve than MX phase, and M_23_C_6_ is the first carbide precipitated at the grain boundary and non eutectic grain boundaries of unstable stainless steel. The addition of niobium and other stabilizing elements can inhibit the precipitation of M_23_C_6_ at grain boundaries. However, the addition of niobium does not exhaust the formation of M_23_C_6_ after quenching from high solution treatment temperature during aging. The second precipitation of M_23_C_6_ can also occur due to the release of carbon from the NbC shift. It can be inferred from the different morphologies of niobium phases and solidification subgrain boundaries in Fig. 2 that the formation of the carbide phase riching the chromium in the location Niobium phase which is directly based on austenite lead to the reduce of the corrosion resisitance of the grain boundary around the carbide phase riching the chromium, and the grain boundary around the chromium rich carbide rapid erosion in the process of metallographic corrosion.

**Table 6:** Chemical composition of Solidification subgrain boundary and austenite intergrain(mass fraction, %).

location	C	Si	Mn	Cr	Ni	Mo	N	Nb	Cu
solidification subgrain boundary	0.052	0.455	4.168	21.207	16.908	1.740	0.135	0.980	5.265
austenitic intergranular	0.026	0.313	2.923	17.040	15.562	0.750	0.140	0.595	3.445

### Solidification grain boundary

**Figure 4:**
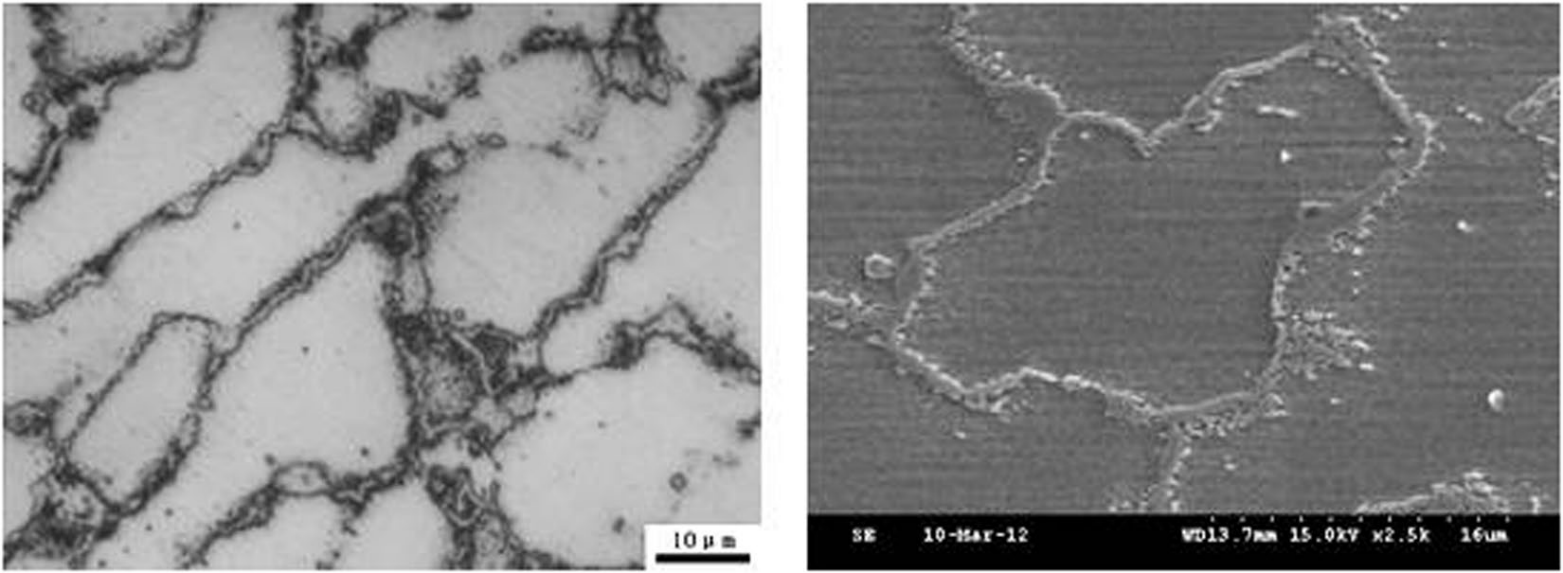
Solidification grain boundary morphology after high temperature enduring tests.

After high temperature enduring tensile, the microstructure morphology of the weld metal solidification grain boundary is shown in Fig. 4. The SEM morphology of the solidified grain boundary shows that it is a chain or cyclic distribution. In the process of high temperature enduring tensile, the discontinuity of chain may be due to the decrease of corrosion resistance, which is easily eroded by corrosive liquid in the process of corrosion.

**Figure 5:**
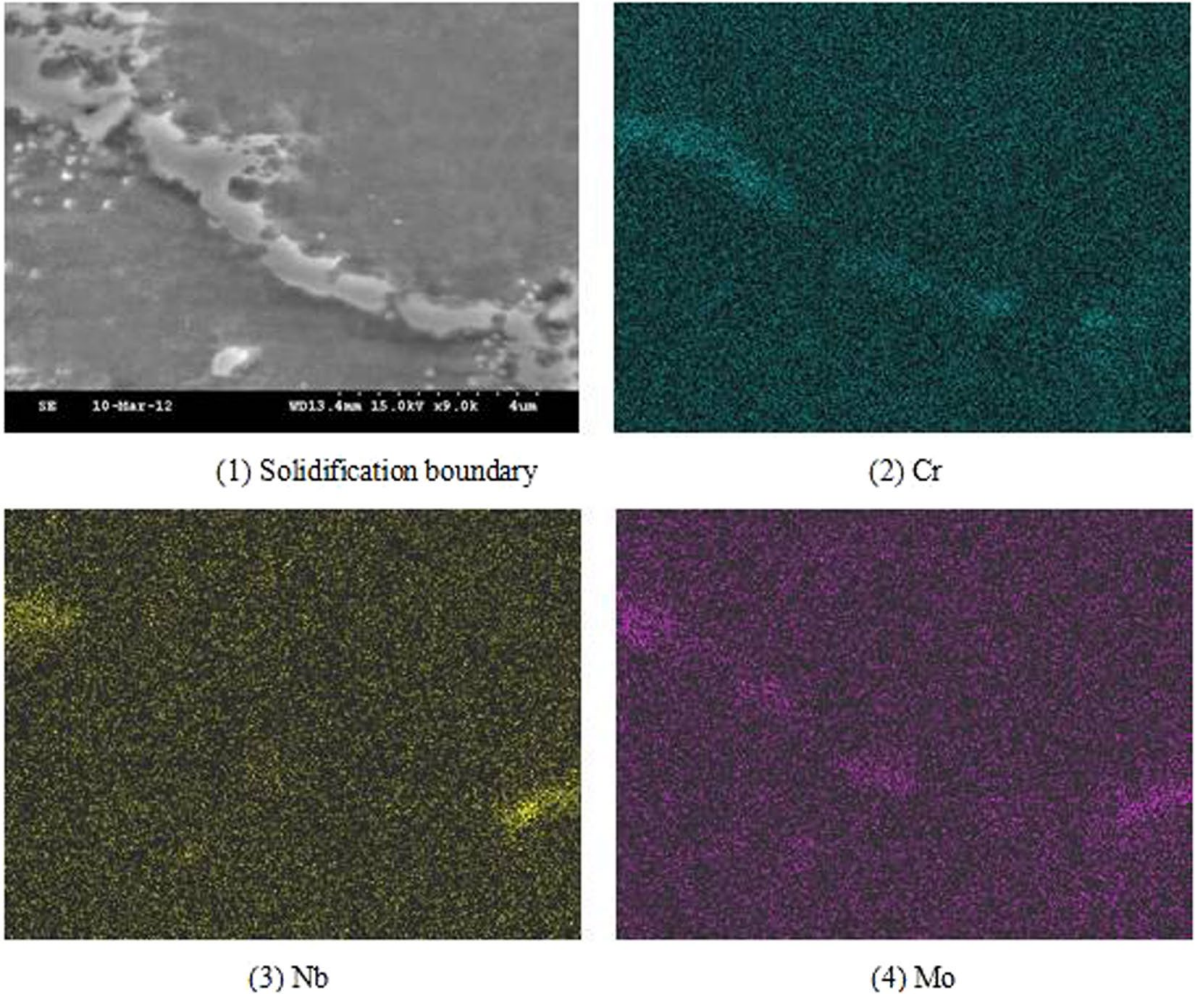
Scanning distribution of grain boundary element.

The results of surface scan analysis of solidification grain boundary are shown in Fig. 5, which shows that the segregation of chromium in the solidification grain boundary is different from that of the solidification subgrain boundary. The energy spectrum analysis showed that the chromium content of the solidification grain boundary was 40%. In addition, the segregation of molybdenum in the solidification grain boundary is also found. It is difficult to distinguish Nb phase and solidification grain boundary under scanning electron microscope from the morphologies of solidification grain boundary in Fig. 5(1). The relative positions of niobium phases and solidification grain boundaries can be identified from the distribution of Nb element in Fig. 5(3).

In addition, the actual tensile stress is not the same due to the different cross-sectional area of the end of the tension specimen and the parallel end. The effective diameters of the clamping end and the parallel end are 8 mm and 5 mm, and the actual stress of the two sections can be obtained from the area ratio. The forces of the clamping end and the parallel end are 200 MPa and 78 MPa. From the observation of the clamping end subjecting the 78 MPa persistent stress and 650 °C high temperature, the microstructure morphology of the solidification subgrain boundary, Nb phase and solidification grain boundary in the clamping end are very close to the parallel end subjecting the 200 MPa persistent stres. It is shown that the microstructure of the deposited metal under different enduring stress has the similar changing tendency under the same the enduring temperature ans time.

### Precipitation of Second phase

**Figure 6:**
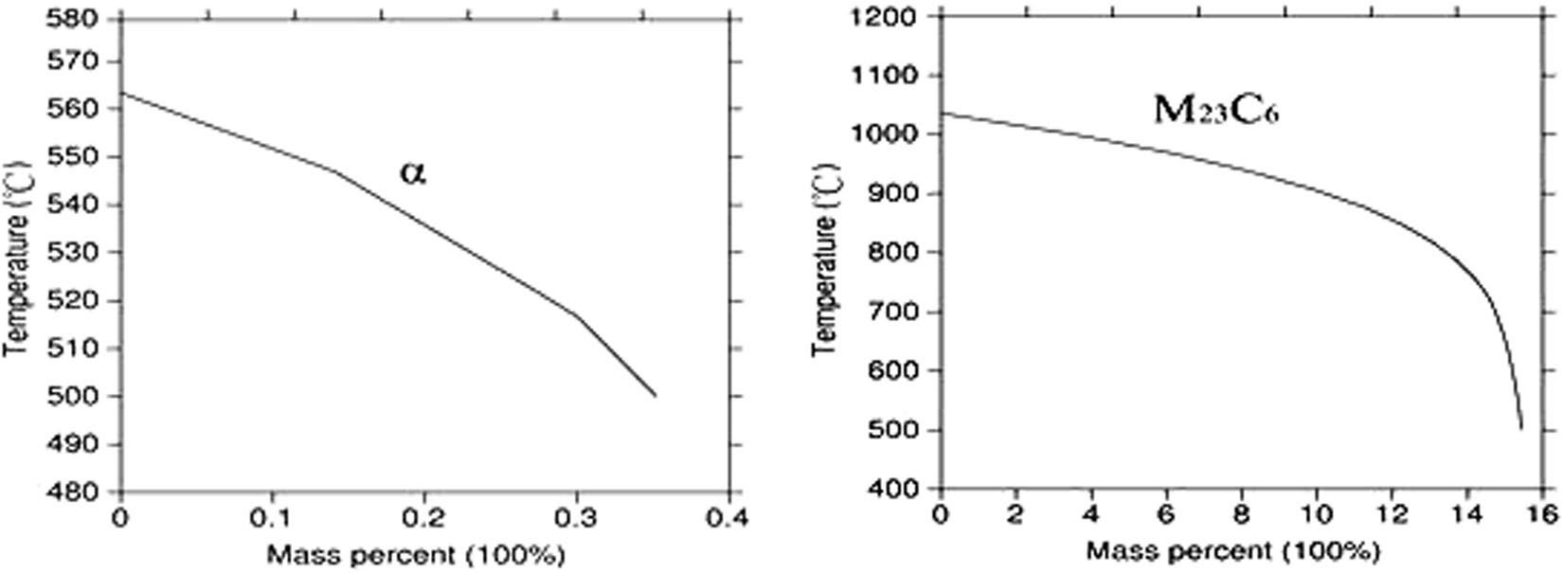
Thermo-calc calculation of the mass fraction of precipitated metal alloy.

According to the Thermo-calc calculation results of the deposited metal alloy system as shown in Fig. 6, the alloy system may also produce α phases and M_23_C_6_ phases in the equilibrium state. The mass fraction and temperature Precipitation curves of γ phases and α phases, part of the γ phases will be transformed into α phases when the temperature decreases to below 570  °C, which is in accordance with the state that the decrease of γ phases is well consistent with the increment of α phases during the temperature range of 600∼500  °C^16^. According to the results of phase analysis in the deposited metal, the M_23_C_6_ and α phases were not precipitated by the rapid cooling of the molten pool. However, the precipitation curves of the M_23_C_6_ and α phases show that the high temperature enduring process is located in the phase precipitation temperature range. Therefore, the possibility of precipitation exists in both the M_23_C_6_ and α phases.

**Figure 7:**
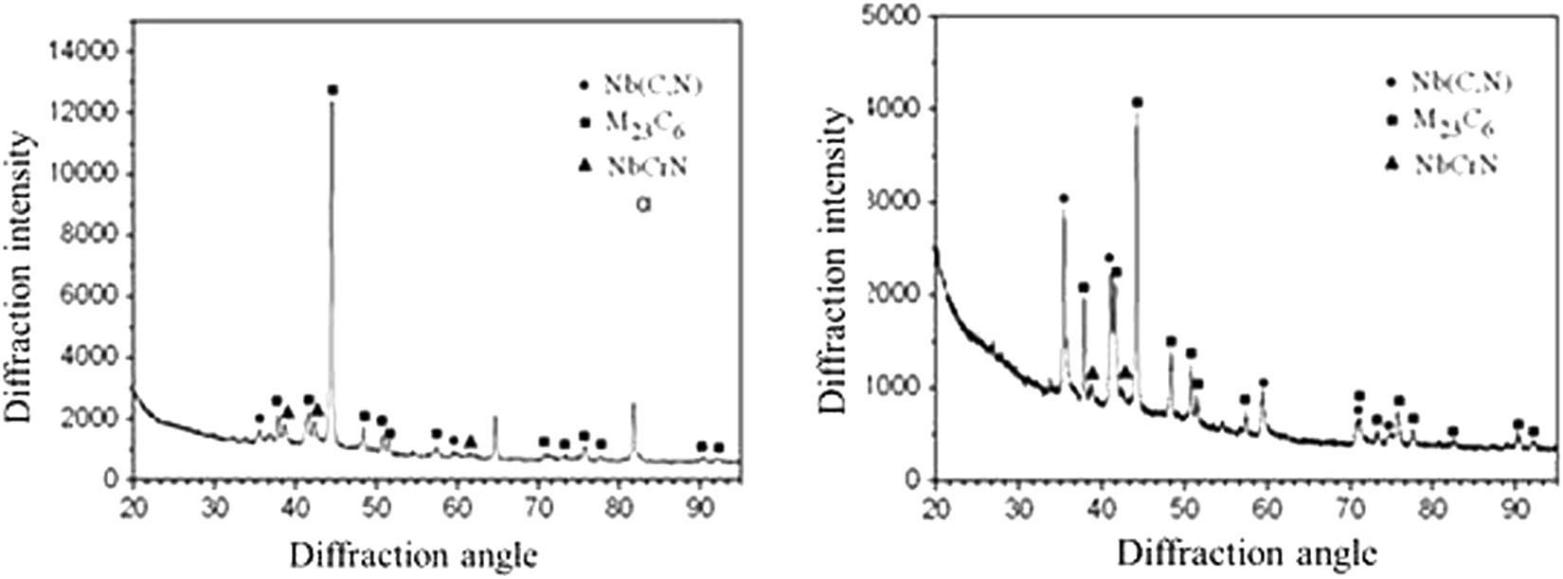
XRD analysis of high temperature deposited metal.

**Figure 8:**
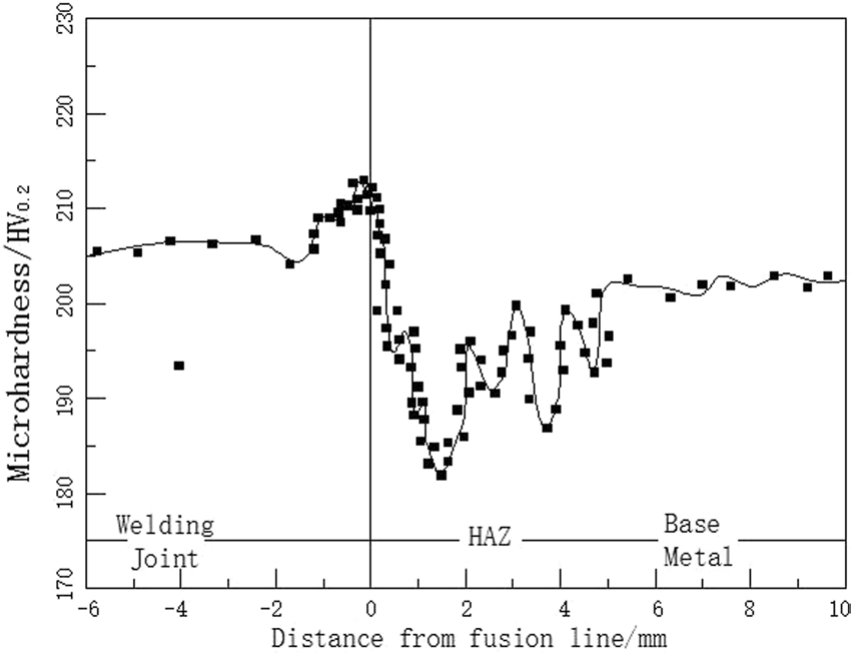
Microhardness distribution of SUPER304H welded joints.

The precipitates in the deposited metal with persistent stress of 200 MPa are mainly Nb (C, N), M23C6 and NbCrN as shown in Fig. 7(2), which is consistent with the calculated results of the precipitated phases in the deposited metal alloy system and the precipitation phases of the Super304H steel. However, there is a certain amount of α phases precipitates in the deposited metal with persistent stress of 78 MPa. There is little difference in the hardness of the austenitic matrix of the deposited metal after welding and high temperature enduring tensile, as shown in Fig. 8, which illustrate that the effect of precipitation strengthening and solid solution strengthening loss of precipitations in the austenite intergrains achieves balance in the process of high temperature enduring tensile, and which also showed that the high temperature strength of deposited metal has high stability at the same time. Because of the size constraints of the long tensile specimen, the results of quantitative analysis of precipitates were not obtained.

As shown in Fig. 8, the hardness distribution of the joint is not uniform, and the hardness of the weld is slightly higher than that of the base metal. The hardness distribution of welding heat affected zone is uneven, the hardness of fusion zone is higher, and the hardness of softening zone is lower. The weld metal structure is a supersaturated solid solution formed by rapid cooling from molten state. The results show that the precipitates in the weld are mainly Nb (C, N) and Cu rich phases. The relative content of Nb (C, N) in the weld is obviously higher than that in the base metal, so the precipitation strengthening effect of the weld is obvious, and the microhardness of the weld is higher than that of the base metal. Due to the semi melting state, a lot of Nb (C, N) precipitates will be produced in the fusion zone accompanied with the solidification process, resulting in precipitation strengthening in the region. The hardness of fusion zone is relatively high, but the softening zone is relatively low because of the dissolution of Cu rich phase and the dissolution of Nb (C, N). The hardness of the joint is about HV200 because of the narrow width of the fusion zone and the softening zone.

#### M_23_C_6_ phases

The precipitation of M_23_C_6_ is controlled by time and temperature, which mainly depends on the diffusion of carbon and the solubility of carbon in the parent material at higher temperature. After aging at 650  °C × 500 h, the precipitation of M_23_C_6_ phases was found in grain boundary and intragranular of the weld seam of Super304H steel^17^. At high temperature, the diffusion of carbon in the austenite matrix of the deposited metal increased, and the elastic stress gradient appear in deposited metal due to the external enduring stress, which will promote the migration of carbon atoms to the elongated part of the lattice of austenite decent and further promote the diffusion of carbon^18,19^. In addition, the diffusion activation energy of grain boundary is only equivalent to the 2/3 of the diffusion activation energy in the matrix. Therefore, the diffusion of carbon on grain boundaries will be further intensified.

Table 6 shows the difference of the content of chromium in the solidification subgrain boundary and austenite matrix. It can be seen that the grain boundary will be the preferential precipitation position of M_23_C_6_. With the increase of the concentration gradient of chromium, a certain degree of chromium deficiency will occur. After the high temperature enduring tensile, the precipitation of M_23_C_6_ in the deposited metal just explained the reason of the different corrosion morphology of the microstructure, especially the interface morphology of the solidification sub grain boundary and Nb phases. In general, the M_23_C_6_ with a cell shape distributed in the grain boundary can hinder the grain boundary sliding, which can increase the lasting strength, while the M_23_C_6_ with a chain shape distributed will lead to embrittlement. In addition, the precipitation of M_23_C_6_ is beneficial to the improvement of creep strength, although molybdenum can stabilize M_23_C_6_, but it is still easier coarsening than MX or Z phases. The substitution of boron to carbon is helpful to stabilize the grain boundary and intergrain of M_23_C_6_ phases, increase the density of M_23_C_6_ carbide, reduce the tendency of coarsening, which is helpful to improve creep performance^20^. Therefore, if the right amount of boron is added to replace the carbon in the weld, the growth of M23C6 can be effectively suppressed, and the lasting strength of the weld can be improved to a certain extent.

#### NbCrN phases

After high temperature enduring tensile test, the NbCrN (Z) phase in the deposited metal has tetragonal structure. NbCrN is usually formed in 347 steel with nitrogen content of more than 0.06%, but it is a secondary phase when the nitrogen content is lower than 0.06%. The NbCrN phase can be formed in a wide temperature range when the nitrogen content is high. Compared with Nb (C, N) phase, the precipitated phase NbCrN is very stable, and NbCrN does not dissolve after solution treatment of 1027  °C × 1 h in the 18%Cr-12%Ni steel with niobium and nitrogen content of 0.3% and 0.09% respectively.

According to the calculation results of Thermo-calc, there is no NbCrN in the precipitated phase of the deposited metal alloy system^13^. It can be seen that NbCrN is only in the high temperature lasting process, and the igh temperature enduring stress can promote the precipitation. The TTP curve of NbCrN in Super304H steel showed that the precipitation time is about 1000 h at the temperature of about 650 °C^21^. Under the condition of 600 °C high temperature and 177 MPa persistent stress, NbCrN phase appears after 85426.7 h^22^. The size of the NbCrN phase is about 1.2μm, and its composition is as following: Nb 23.7%, Cr 53.7%, Ni 5.8%, Cu 5.7%, Fe 10.7%. The NbCrN phases can be precipitated from the 18%Cr-12%Ni steel containing nitrogen and niobium after long-term insulation at the temperature of 800–850 °C, and and its composition is as following: Nb 44.6%, Cr 22.9%, Mo 3.8%, N 6.4%, Fe 5.1%, Mn 2.2%, C 0.2%. It can be seen that the total amount of chromium and niobium in NbCrN exceeds 65%. The precipitation of NbCrN phases in the deposited metal will consume a large amount of niobium and chromium from the basic composition of the NbCrN phase. The grain boundaries of the solidification of the grain boundaries, which are relatively enriched in nitrogen, chromium and niobium, as well as the grain boundaries of the segregation of chromium, will be the preferential precipitation sites. In addition, the partial dissolution of niobium phase at the sub grain boundaries can provide niobium for the formation of the phase. According to YahongYang *et al.*^23^, the secondary NbCrN and M_23_C_6_ are the predominant precipitates during creep. Secondary NbCrN is the most important strengthening precipitate of S31042 steel. At least 75% of the precipitation hardening results from secondary NbCrN. Furthermore, secondary NbCrN is the main factor to affect the variation of hardness of SUPER304H steel during creep. So, it can be concluded that the NbCrN phase precipitates at the interface between the solidification grain boundary and the Nb phase. The precipitation of NbCrN will further deplete the chromium element in the grain boundary and the austenite matrix, which is not conducive to the overall corrosion resistance of the deposited metal. However, it will help to improve the lasting strength of deposited metal due to the high temperature stability of the phase.

#### α phases

A certain amount of α phase was found in a durable stress of 78 MPa at the clamping end of the deposited metal. The calculation of the equilibrium state of the deposited metal alloy system shows that a certain amount of α phase is formed in the alloy system at the temperature of 563 °C. It is obvious that the possibility of the formation of α phase exists in the alloy system. This phase is mainly composed of iron and chromium, of which the proportion of iron is above 80%. With the decrease of temperature, the proportion of iron in the α phase will be further increased. As for austenitic steel weld metal with chromium content close to 17%, there is no report on the appearance of alpha phase after the high temperature enduring tensile test at the temperature of 650  °C.

Generally speaking, the alloy with the the chromium content exceeding 14% had the tendency to generating the ferrite α riching iron element and the ferrite α′ riching chromium element, but the tensile temperature is not in this temperature range^24^. It can be seen from the composition of the α phases that the high content of iron is beneficial to its formation. The statistical analysis of the composition of the different micro regions showed that the iron content in the austenite matrix was higher than that of the grain boundary, the carbon content was 0.026%, and the nickel content was more than 15%. Nb (C, N) phases may be precipitated in austenite grains during the high temperature enduring tensile tests. With the precipitation of the phase, the locations surrounding Nb (C, N) phases will occurs the relatively poor carbon and nitrogen elements. The decrease of the content of the elements is beneficial to the formation of the α phases. If the iron and chromium elements gather in the vicinity of Nb (C, N) phase, the alpha phase may be generated. Therefore, the interface between the austenite matrix and the Nb (C, N) of the austenite matrix will be the favorable position of the alpha phase nucleus. From the results of XRD analysis of deposited metal under different stress, no α phase was found in the deposited metal with persistent stress of 200 MPa. If the interference caused by the accuracy of the phase analysis test is not considered, the α phase in the deposited metal can be produced under certain stress conditions at the same temperature and duration. The higher stress can accelerate the diffusion of elements, which may change the precipitation kinetics of α phase.

Due to the low corrosion resistance of iron rich phase, the corrosion resistance of the structure will be reduced when it appears with the high content of chromium and nickel. In addition, as for the Super304H heat resistant steel weld under high temperature and lasting conditions, the existence of α phase will promote the formation of σ brittle phase, so it is necessary to inhibit the formation of α phase in the process of high temperature. Therefore, it is necessary to improve the content of carbon, nitrogen and other elements of austenite to inhibit the precipitation of α phase. It can be seen that when the carbon content of deposited metal is 0.08%, it is difficult to inhibit the appearance of α phase. However, the increase of carbon content will affect the corrosion resistance, so the nitrogen content could be increased properly without affecting the precipitation of Nb (C, N).

In addition, due to the limitations of the detection methods, the presence of copper rich phase in the deposited metal can not be found after the high temperature, and the precipitation of copper in the deposited metal should be further studied.

#### Effect of phase separation on corrosion resistance

**Figure 9:**
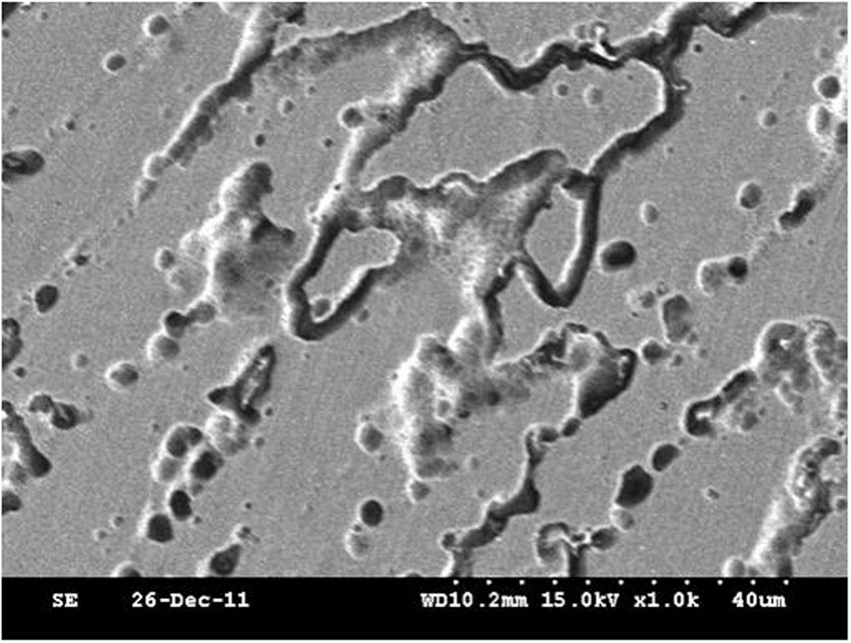
Electrolytic corrosion morphology of high temperature durable specimens.

The same electrolytic etching method was used for the corrosion resistance of the permanent sample as the same as that of the welding sample. After corrosion, a large number of holes appear in the high temperature lasting tissues, as shown in Fig. 9. Compared with the tissue morphologies after the chemical corrosion, the pits in the high temperature lasting tissues are the morphologies of the precipitates, the solidification subgrain boundary and the solidification grain boundary falling off from the matrix, and can the residual Nb phases also can also be detected in the part of cavity. It is shown that the corrosion resistance of the tissues after 8960 h enduring tests was significantly reduced compared with the deposited metal. In addition, the smaller size of the holes can also be seen in the austenite matrix. It can be inferred that the bonding strength of the precipitated phase, the grain boundary and the austenite matrix in the deposited metal decreases after the high temperature tensile test.

Due to the enrichment of alloying elements in the solidification sub grain boundaries and solidification grain boundaries, Nb (C, N), NbCrN and M_23_C_6_ precipitates are easy to precipitate, which consumes a large amount of chromium and leads to a further reduction of the corrosion resistance for the grain boundary. The voids in austenite grains are likely to be the precipitation position of Nb (C, N) and α phases. The precipitation of precipitated phase decreases the homogeneity of austenite microstructure. Under the action of corrosive medium, the continuity of the precipitates and the solidification subgrain boundary, the solidification grain boundary and the matrix will decrease, and the interface is likely to be the nucleation of cracks^25^. The crack at the interface will become the weak link and eventually lead to the failure of the joint because of the action of persistent stress. The corrosion resistance and endurance strength of deposited metal has the inversely proportional relationship. Therefore, it is necessary to consider the influence of the number and size of the precipitated phases in the design of Super304H steel weld, and to determine the content of alloy elements on the basis of the comprehensive corrosion resistance and lasting strength.

## Conclusions

1. The high temperature tensile specimen is not broken after 8960 h. The Nb phases in the location without affecting by the welding two-thermal cycle dissolve, and there is no obvious diffusion of Nb and Mo.
2. There exists the segregation of Cr and Mo in the grain boundary, and the chromium content is up to 40%. The corrosion resistance is relatively high after the high temperature tensile strength, and it is difficult to distinguish the metallographic corrosion morphology and niobium phase.
3. With the persistent stress of 200 MPa, the precipitated phase of deposited metal is Nb (C, N), M_23_C_6_, NbCrN phase, and a certain amount of α phase is precipitated in the deposited metal with a persistent stress of 78 MPa. The precipitation of M_23_C_6_ phase is the main reason for the decrease of the corrosion resistance.

## Electronic supplementary material

Supplementary File
