# Supplementary material for: Microstructure and phases of deposited metal of SUPER304H steel under high temperature Persistent stress
Source: Sci Rep. 2018 Feb 8;8:2618. doi: 10.1038/s41598-018-20594-9 (PMC5805682; doi:10.1038/s41598-018-20594-9)
Supplement: Supplementary file 1 — Supplementary File [file 41598_2018_20594_MOESM1_ESM.pdf]

Microstructure and phases of deposited metal of SUPER304H steel under high temperature Persistent stress

QI Yanchang[1], WU Zhiquan[2], ZHANG Xin[3*], MA Chengyong[1]

1. Welding Research Institute, Central Iron and Steel Research Institute, Beijing 100081, China

2. Anhui Branch, China Datang Coporation, Anhui Hefei 230088, China

3. Institute of Thermal Power Generation Technology, China Datang Coporation Science and Technology Research Institute, Beijing 102206, China

1.Introduction

Due to the high degree of supersaturation after welding of Super304H [11], there was more obvious reduction in the aging process, and there are some differences between the factors of aging embrittlement and the base metal. Therefore, it is necessary to study the cause of aging embrittlement. According to the published literature, the research mainly focused on the properties and strengthening mechanism of Super304H steel base material and the selection of the welding material [12].

On the basis of the study of SUPER304H welding wire in the earlier period[13], this paper has studied the microstructure and phases of deposited metal of SUPER304H steel under high temperature persistent stress in order to master the related properties of welding wire.

2. Experimental materials and methods

2.1 Experimental materials

The diameter of the welding wire was 1.6mm. The actual compositions of the test welding wires were determined by ICP-AES analysis and the results were presented in Table 1. The test wire was used to deposite the cladding isolation layer with thickness of 8 mm on the groove surface and the pad surface, and the root clearance was 16 mm, which ensured that the deposited metal component was not affected by the dilution of the substrate. The SUPER304H austenitic heat-resistance steel is used for the test, and the composition of SUPER304H steel tube was shown in Table 2.

Table 1 Chemical composition of deposited metal (mass fraction,%)

Element	C	Si+Mn	Cr	Ni	Mo	N	Nb	Cu
content	0.080	3.39	16.97	15.24	1.09	0.13	0.28	2.91

Table 2 Chemical composition of Super304H (mass fraction，%)

C	Si	Mn	S	P	Cr	Ni	Mo	N	Nb	Cu	Co	Al

* Foundation Item: Supported by China Datang Coporation
Corresponding author. ZHANG Xin, E-mail: kmzx201@163.com, +86-10-80732928

0.074	0.28	0.81	0.003	0.016	18.26	9.01	0.32	0.11	0.53	2.95	0.031	0.016

2. 2 Experimental methods

Welding equipment adopted Manipulator AMET welding machine automatic TIG system. Welding heat input is 14 KJ/cm, and the specific welding process parameters are shown in Table 3.

Table 3 welding process parameters of welding wire deposited metal

Electric current/A	Voltage/V	Welding speed cm/min	Wire feeding speed mm/min	shielding gas	Gas flow L/min	Inter channel temperature ℃
260	12.7	14	800	Pure Ar	15	≤100

The test pressures were 200 and 78 MPa at the temperature of 650℃. The sample spans SUPER304H weld and the center of the sample is the weld center. After grinding and polishing, the specimen was etched by the mixed solution consists of 5g $CuCl_2$, 30 ml HCl, 25 ml alcohol and 30 ml H_2O. Then，the microstructure of deposited metal was investigated by using the MEF-4M metallographic microscope and SCIAS 6.0 image analysis system. The second phase of deposited metal and impact fracture surface were analyzed using Scanning Electron Microscope of HITACHI S-4300 with matching Energy Dispersive Spectrometer.

In order to confirm the possible precipitated phase, the method of electrochemical extraction was used to study the second phase of deposited metal. In addition, X Pert Pro X-ray diffractometer was also adopted for the analysis of phase composition of deposited metal.

References

[1] F Msuyama. History of Power Plants and Progress in Heat Resistant Steels. *ISIJ INT*. **2**(4), 199-206(2007).

[2] YS Yi, Y Watanabe, T Kondo, H Kimura, M Sato. Oxidation Rate of Advanced Heat-Resistant Steels for Ultra-Supercritical Boilers in Pressurized Superheated Steam. *J PRESS VESS-T ASME*. **123** (3): 391-397(2001).

[3]Takao K, Sawaragi Y. Properties atter service exposure of a new new austenitic stainless steel Super304H(0.1C-18Cr-9Ni-3Cu-Nb，N) for boiler tube application. *Sumitomo Search*. **10**: 45-48(1993).

[4] Shuping Tan, Zhenhua Wang, Shichang Cheng, Zhengdong Liu, Jiecai Han, Wantang Fu. Processing maps and hot workability of Super304H austenitic heat-resistant stainless steel. *MAT SCI ENG A*. **517**(1-2): 312-315(2009).

[5] Ruikun Wang, Zhijun Zheng, Qinwen Zhou, Yan Gao. Effect of surface nanocrystallization on the sensitization and desensitization behavior of Super304H stainless steel. *CORROS SCI*. **111**: 728-741(2016).

[6]Sawaragi Y, Ogawa K, Kato S. Development of the economical 18-8 stainless steel (Super304H) having high elevated temperature strength for fossil fired boillers. *The Sumitomo Search*. **48**(1): 50-58(1992).

[7]Sawaragi Y, Hirano S. The development of a new 18-8 austenitic steel (0.1C-18Cr-9Ni-3Cu-Nb, N) with high elevated temperature strength for fossil fired boilers. *USA：New Alloys for Pressure Vessels and Piping.* 141-146(1990).

[8] Y Noguchi，M Miyahara，H Okada，M Igarashi. Effect of grain size on creep-fatigue properties of 18Cr-9Ni-3Cu-Nb-N steel under uniaxial and torsional loading. *J Soc Mater Sci*. **56**(2): l36-141(2007).

[9] Myung-Yeon Kim, Suk-Chul Kwak, In-Suk Choi, Young-Kook Lee, Jin-Yoo Suh, Eric Fleury, Woo-Sang Jung, Tae-Ha Son. High-temperature tensile and creep deformation of cross-weld specimens of weld joint between T92 martensitic andSuper304H austenitic steels. *MATER CHARACT*. **97**: 161-168(2014).

[10] Y Sawaragi, S Hirano. TS5c2–The Development of a New 18-8 Austenitic Stainless Steel (0.lC-18Cr-9Ni-3Cu-Nb, N) with High Elevated Temperatures Strength for Fossil Power Boilers. *Mechanical Behaviour of Materials VI*. 589-594(1992).

[11]Xin-mei Li, Yong Zou, Zhong-wen Zhang, Zeng-da Zou. Microstructure Evolution of a Novel Super304H Steel Aged at High Temperatures. *MATER TRANS*. **5l**(2): 305-309(2010).

[12] A Yaeina, V Souza, S Tavares, J Pardal, J Souza. Microstructure and intergranular corrosion resistance evaluation of AISI 304 steel for higll temperature service[J]. *MATER CHARACT*. **59**(5): 651-655(2008).

[13] QI Yanchang, WU Zhiquan, ZHANG Xin, MA Chengyong. Microstructure and properties of deposited metal of SUPER304H steel. *SCI REP-UK*, 14454(7): 1-13 (2017).

[14] A.Iseda, H.Okada, H.Semba, M.Igarashi. Long term creep properties and microstructure of SUPER304H, TP347HFG and HR3C for A-USC boilers. *ADV ENERGY MATER*. **2**(4): 199-206(2007).

[15] B.R. Clark. The shape and mechanism of formation of $M_{23}C_6$ carbide in austenite. *Acta Metall.* **15**: 113–129(1967).

[16]Yinghui Zhou, Yongchang Liu, Xiaosheng Zhou, Chenxi Liu, Jianxin Yu, Yuan Huang, Huijun Li, Wenya Li. Precipitation and hot deformation behavior of austenitic heat-resistant steels: A review. *J MATER SCI TECHNOL*(In Press).

[17] Yanhui Li, Shuzhong Wang, Panpan Sun, Zhenxia Tong, Donghai Xu, Yang Guo, Jianqiao Yang. Early oxidation of Super304H stainless steel and its scales stability in supercritical water environments. *INT J HYDROGEN ENERG*. **41**(35): 15764-15771(2016).

[18] Yong Qilong. Secondary phase in steel. *Beijing: Metallurgical Industry press*. 189-192(2006).

[19] Dudziak T., Deodeshmukh V., Backert L., Sobczak N., Witkowska M., Ratuszek W., Chruściel K., Zieliński A, Sobczak J., Bruzda G.. Phase Investigations Under Steam Oxidation Process at 800°C for 1000 h of Advanced Steels and Ni-Based Alloys. *OXID MET*. **87**(1-2): 139-158(2017).

[20] P. Ou, H. Xing, X.L. Wang, J. Sun. Tensile yield behavior and precipitation strengthening mechanism in Super304H steel. *MAT SCI ENG A-STRUCT*. 600(10): 171-175(2014).

[21] ZW Zhang, LI Xin-Mei, Y Zou, DU Bao-Shuai. Microstructure of Super304H Steel after Aging at 650℃ for Different Times. *MATER MECH ENG*. **35**(11): 5-10(2011).

[22] Zieliński A., Golański G., Sroka M., Dobrzański J.. Estimation of long-term creep strength in austenitic power plant steels. *MATER SCI TECH-LOND*. **32**(8): 780-785(2016).

[23] YahongYang, LihuiZhu, QijiangWang, ChangchunZhu. Microstructural evolution and the effect on hardness and plasticity of S31042 heat-resistant steel during creep. *MAT SCI ENG A-STRUCT*. **608**(25) : 164-173(2014).

[24] Guanshun Bai, Shanping Lu, Dianzhong Li, Yiyi Li. Influences of niobium and solution treatment temperature on pitting corrosion behaviour of stabilised austenitic stainless steels. *CORROS SCI*. **108**: 111-124(2016).

[25] Dae-Bum Park, Sung-Min Hong, Kyu-Ho Lee, Moo-Young Huh, Jin-Yoo Suh, Seung-Cheol Lee, Woo-Sang Jung. High-temperature creep behavior and microstructural evolution of an 18Cr9Ni3CuNbVN austenitic stainless steel. *MATER CHARACT*. **93**: 52-61(2014).
